# A Moexitecan Magnetic Liposomal Strategy for Ferroptosis-Enhanced Chemotherapy

**DOI:** 10.3390/pharmaceutics15072012

**Published:** 2023-07-24

**Authors:** Weiling Miao, Yang Liu, Jian Tang, Tiandong Chen, Fang Yang

**Affiliations:** State Key Laboratory of Digital Medical Engineering, Jiangsu Key Laboratory for Biomaterials and Devices, School of Biological Sciences and Medical Engineering, Southeast University, Nanjing 210096, China; weilling_miao@seu.edu.cn (W.M.); lyseubme@seu.edu.cn (Y.L.); jiantang@seu.edu.cn (J.T.); 230218251@seu.edu.cn (T.C.)

**Keywords:** moexitecan, liposomes, superparamagnetic iron oxide nanoparticles, antitumor effect, ferroptosis

## Abstract

Moexitecan (Mex) is a novel camptothecin derivative that retains the potent antitumor properties of camptothecin drugs and has improved hydrophilicity to enhance biocompatibility in vitro. However, single-drug therapy still has limitations. In this study, magnetic liposomes loaded with both moexitecan and superparamagnetic iron oxide nanoparticles (SPIO) have been fabricated by a film hydration and filtration method, which is abbreviated as Mex@MLipo. By using liposomes as drug carriers, Mex can be delivered specifically to the target site, resulting in improved therapeutic efficacy and reduced toxicity. Morphology characterization results show that Mex@MLipo has a mean diameter of 180–200 nm with a round morphology. The loading efficiencies of Mex and SPIO are 65.86% and 76.86%, respectively. Cell toxicity, in vitro cell uptake, and in vivo fluorescence imaging experiments showed that Mex@MLipo was the most effective in killing HT-29 cells compared with HepG-2 and PC-3 cells, due to its ability to combine chemotherapy and induce ferroptosis, resulting in a strong anti-tumor effect. Thus, this study developed an innovative nanoscale drug delivery system that paves the way for clinical applications of moexitecan.

## 1. Introduction

Camptothecin, originally extracted from Camptotheca, a traditional Chinese medicine, is a pentacyclic alkaloid that exhibits potent anti-tumor ability [1]. In order to decrease its high toxicity and increase its poor solubility in water, researchers have developed numerous camptothecin derivatives, based on the mechanism of the structure–activity relationship, structural modification, and pharmacodynamics [2,3]. Among them, moexitecan (Mex) is a novel camptothecin derivative synthesized via a structural molecular hybridization and prodrug design [4]. The wrapped Mex enters the nucleus and binds to the replicating DNA and topoisomerase I (Top I) to form a ternary complex that blocks DNA replication and induces apoptosis [5]. In vitro studies have shown that Mex exhibits a significant inhibition of cell proliferation against human ovarian cancer cells [6] and lung cancer cells [7]. Furthermore, Mex demonstrates superior antitumor efficacy compared to the approved drug irinotecan, used against human colon cancer cells [8] and liver cancer cells [9], making it a promising candidate for further clinical development.

In recent years, drug-delivery vehicles have garnered significant attention for overcoming the challenges faced by single-drug therapy, including the inability to precisely target tumors and the potential for systemic toxicity despite the reduced toxicity of the drugs [10]. The development of drug-delivery vehicles can help mitigate these challenges by precisely transporting the drug to the tumor site, reducing the drug amount to mitigate toxic side effects, and enhancing the drug efficacy [11]. Liposomes, ultrafine spherical carriers that encapsulate drugs in lipid bilayers, have emerged as a promising drug delivery system, due to their good biocompatibility, low toxicity, easy biodegradation, and ability to protect drugs from immune system destruction [12,13]. They are of great potential for clinical drug-delivery development [14].

The presence of superparamagnetic iron oxide nanoparticles (SPIO) may inhibit enzymes from the thioredoxin reductase family, preventing the regeneration of intracellular antioxidants and affecting the management of oxidative stress and the content of reactive oxygen species (ROS) [15,16]. ROS are metabolic byproducts generated in eukaryotic cells during aerobic respiration. Their expression is significantly higher in tumor cells than in normal cells. The upregulation of ROS production can activate tumor-promoting pathways that lead to cancer progression, angiogenesis, and metastasis [17]. However, a sustained increase in ROS levels can also induce apoptosis in tumor cells, serving as a potential strategy for cancer therapy [18]. Several studies have suggested that ferroptosis, a form of programmed cell death, is involved in tumor pathogenesis [19,20,21]. Unlike apoptosis, necrosis, and autophagy, ferroptosis has distinct mechanisms. It has been reported that ROS plays a critical role in ferroptosis in prostate cancer where excess Fe^3+^ ions enter cells, are reduced to Fe^2+^ by STEAP3, and accumulate in the unstable Fe^2+^ form [22]. The generated hydrogen peroxide, in the presence of Fe^2+^, produces strong oxidizing hydroxyl radicals and other ROS that catalyze the peroxidation of unsaturated fatty acids on cell membranes, promoting ferroptosis and inhibiting prostate cancer growth [23].

**Figure 1:**
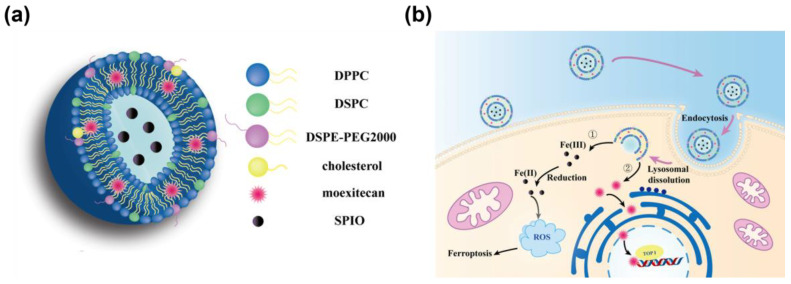
Design features and mechanisms of Mex@MLipo for effective antitumor activities. (**a**) A stereogram of Mex@MLipo shows that the phospholipid bilayer consists of DPPC, DSPC, DEPE-PEG2000, and cholesterol. Mex is embedded in the bilayer, and magnetic nanoparticles are encased in the hydrophilic core of the liposome. (**b**) Mex@MLipo enters the cytoplasm through endocytosis and is decomposed by lysosomes. The materials loaded into the liposome acts on the cell in two ways. ➀ The magnetic nanoparticles are reduced to Fe^2+^, resulting in more reactive oxygen species production to induce ferroptosis for enhanced antitumor efficacy. ➁ The released Mex enters into the nucleus and binds to the replicating DNA and Top I to form a ternary complex that blocks DNA replication and induces apoptosis.

The aim of this study was to prepare magnetic liposomes loaded with Mex within the lipid bilayer and γ-Fe_2_O_3_ magnetic nanoparticles in the core of the liposome’s structure (Mex@MLipo, Figure 1a). Its antitumor effect for three types of cancer cells (HT-29, HepG-2, and PC-3) has been studied in mouse subcutaneous tumor model. In particular, as illustrated in Figure 1b, the synergistic effect based on ferroptosis induced by SPIO intracellular delivery and Mex chemical killing have been investigated. The results show that the HT-29 cell is the most sensitive for Mex@MLipo, with an enhanced antitumor efficacy. Such drug-loaded magnetic liposomes have great potential for enhanced antitumor and magnetic resonance imaging uses in clinic treatment.

## 2. Materials and Methods

### 2.1. Materials and Agents

The γ-Fe_2_O_3_ superparamagnetic iron oxide nanoparticles were synthesized and modified with poly-glucose sorbitol carboxymethyl ether (PSC), which was provided by the Jiangsu Key Laboratory for Biomaterials and Devices (China) [24]. 1,2-dipalmitoyl-sn-glycero-3-phosphocholine (DPPC), 1,2-distearoyl-sn-glycero-3-phosphocholine (DSPC), and 1,2-distearo-yl-sn-glycero-3-phosphoethanolamine-N-[(carboxyl (poly-ethylene glycol) 2000] (ammonium salt) (DSPE-PEG2000) were purchased from Avanti Polar Lipids Inc. (Alabaster, AL, USA). Mex was provided by Zhengda Tianqing Pharmaceutical Group Co., LTD (Nanjing, China). Trichloromethane and methanol were purchased from Shanghai Chemical Reagent Company (Shanghai, China). 3,3′-dioctadecyloxacarbocyanine perchlorate (DIO), Hoechst 33342, reactive oxygen species detection kit, and enhanced mitochondrial membrane potential test kit (JC-1) were purchased from Beyotime (Shanghai, China). Human colon cancer cells HT-29, human liver cancer cells HepG-2, and human prostate cancer cells PC-3, McCoy’s 5A medium, MEM (containing NAEE) medium, and Ham’s F-12K medium were purchased from Wuhan Procell Life Technology Co., LTD (Wuhan, China). Fetal bovine serum, penicillin/streptomycin, and trypsin-EDTA were purchased from KeyGen Biotechnology Co., LTD (Nanjing, China).

### 2.2. Fabrications of Mex@MLipo

The phospholipid membrane material DPPC (56 μmol), DSPC (14 μmol), DSPE-PEG2000 (2 μmol), cholesterol (7.2 μmol) and Mex (5.3 μmol) were dissolved to 5 mL trichloromethane solution inside a flask. The vacuum environment was performed to remove trichloromethane and form a membrane (50 °C, 90 rpm, 2 h). After film formation, the flask was placed into vacuum drying oven and kept overnight. Then, in order to maintain the anti-tumor activity of Mex, a total of 3 mL 5% glucose solution with SPIO (600 μg mL^−1^, 60 μL) was added in the flask under 60 °C temperature and suspension rotation (72 rpm, 40 min). Then, the mixed suspension was transferred to liposome extruder containing polycarbonate films (pore size: 400 nm). After repeated extrusion for 30 times at a constant rate in a 60 °C oven, the Mex@MLipo was obtained. As for the control, the Mex@Lipo with no SPIO loading was prepared using similar methods to the Mex@MLipo just without SPIO addition in the glucose solution.

To obtain pure Mex@MLipo, the preliminary samples were filtered and purified by dextran gel chromatography. First, 4 g of dextran dry gel was added into 100 mL deionized water and swelled overnight. Small particles and bubbles in the gel suspension were removed by heating and stirring. Next, a hollow tube was gently and slowly filled with 9 mL of the gel column. Then, the gel column was centrifuged for 5 min at 1300 rpm to remove excess ultra-pure water. To purify the liposome samples, 900 μL liposome samples was dropped onto the top of the gel column, centrifuged, and eluted for 5 min at 1300 rpm. At last, eluent was collected. This process was repeated for 3 times, and the Mex@MLipo eluent was collected in a centrifuge tube and stored at 4 °C before the experiment.

The Mex@Lipo was purified by using the same method as the Mex@MLipo.

### 2.3. Characterization of Mex@MLipo

Surface morphology was determined by a Transmission Electron Microscope (TEM) (JEM-2100, JEOL, Tokyo, Japan). In brief, liposome samples (10 µL) were dropped onto copper 400-mesh grids. After draining via a filter paper for 30 min, a phosphotungstic acid stain solution (1.5% by weight, adjusted to pH 6.0) was applied for 10 min, and TEM images were taken. The hydrodynamic sizes and polydispersity indices (PDIs) of Mex@MLipo and Mex@Lipo were measured using a Zeta-Sizer Nano-ZS 90 (Malvern Instruments, Malvern, UK). The zeta potential of the formulations was determined after a tenfold dilution by laser Doppler velocimetry using a Nanosizer ZS with a universal dip cell (Malvern Instruments, UK). Each sample was measured in triplicate.

### 2.4. Stability Evaluation of Mex@MLipo

To further evaluate the physical stability of Mex@MLipo, the particle size and surface zeta potential of the liposomes were measured and recorded on days 1, 5, 9, 13, 17, 21, 25, and 29 after preparation when stored at 4 °C.

### 2.5. FT-IR Characterization of Mex@MLipo

First, 1 mL solutions of Mex@MLipo and Mex@Lipo were freeze-dried for 48 h to obtain sample powder, respectively. The sample powder and Mex were characterized by a Fourier transform infrared (FT-IR) spectrophotometer (IRAffinity-1, Shimadzu Corporation, Kyoto, Japan). The wavenumber range from 4000 cm^−1^ to 400 cm^−1^ was scanned for 64 repeats, and the scanning process was repeated 3 times.

### 2.6. Vibrating Sample Magnetometer Characterization of Mex@MLipo

To analyze the magnetic response performance of Mex@MLipo, the hysteresis lines of Mex@MLipo and SPIO were measured using a vibrating sample magnetometer (VSM). In total, 20 mg of freeze-dried powders of Mex@MLipo and SPIO were weighed separately, and the weighing paper wrapped around the samples was folded to a size of 0.5 × 0.5 cm and placed inside the measurement chamber for measurement.

### 2.7. Measurement of Mex Encapsulation Efficiency of Mex@MLipo

To confirm the insertion of Mex in the liposome membranes, and to quantitatively measure the concentration of Mex loaded in the liposomes, an ultraviolet-visible (UV-vis) spectrophotometer (UV-3600, Shimadzu, Japan) was employed. Firstly, a standard Mex–methanol solution ranging from 0 to 100 μg mL^−1^ was prepared and scanned for the full ultraviolet spectrum. Specific absorption peaks of the Mex–methanol solution were identified. A concentration–absorbance standard curve of drugs in pure methanol solution was plotted to obtain the relationship equation. Next, the liposomes were disintegrated using methanol solution overnight. The disintegrated liposome sample was scanned by UV-vis spectroscopy at a specific wavelength. The concentration of Mex in methanol solution was deduced backwards according to the standard curve, and the encapsulation efficiency of Mex in Mex@MLipo was obtained.

### 2.8. Measurement of Iron Encapsulation Efficiency of Mex@MLipo

To quantitatively measure the concentration of SPIO loaded in the Mex@MLipo, the iron element was calculated using a UV-vis spectrophotometer after gel column purification. Firstly, the national standard solution of iron element was taken, and the gradient solution with iron concentration of 0–10 mg L^−1^ was prepared to obtain the standard curve of iron ion concentration–absorbance. To obtain the iron encapsulated in the liposome, a 2 mL 6 M HCl solution and a 1 mL hydroxylamine hydrochloride solution were added, sonicating for 5 min to reduce Fe^3+^ to Fe^2+^. Then, 2 mL of phenanthroline solution, 2 mL of 6 M NaOH solution, and 5 mL of HOAc-NaAc solution were added. After the color change reaction, absorption value of resulting sample was measured at wavelength of 510 nm by UV-vis spectroscopy. The encapsulation efficiency of iron in Mex@MLipo was obtained.

### 2.9. Cell Lines and Cell Culture

HT-29 cells were maintained in McCoy’s 5A medium supplemented with 10% FBS and 1% penicillin/streptomycin. HepG-2 cells were maintained in MEM (containing NAEE) medium supplemented with 10% FBS and 1% penicillin/streptomycin. PC-3 cells were maintained in Ham’s F-12K medium supplemented with 10% FBS and 1% penicillin/streptomycin. Cells were cultured in 25 cm^2^ sterile tissue culture flask at 37 °C and 5% CO_2_ level. Cells were passaged twice a week using trypsin-EDTA when reaching 80% confluency.

### 2.10. Cytotoxicity of Mex@MLipo

The cytotoxicity values of Mex@MLipo for HT-29, HepG-2, and PC-3 cells were determined via CCK-8 cell proliferation assay based on a modified manufacturer’s protocol. Briefly, HT-29, HepG-2, and PC-3 cells were seeded with 96-well plates at a density of 5 × 10^3^ cells per well and were cultured overnight, followed by the addition of the Mex@MLipo at determined concentrations (equivalent to 0, 10, 20, 30, 40, and 50 µg mL^−1^ of Mex). After an additional 24 h incubation, CCK-8 solution (10 µL) in medium (90 µL) was added to each well and incubated for another 1.5 h. The absorbance intensity in each well was measured at 450/650 nm by using a multimode microplate reader Infinite M200 PRO (Tecan instruments, Raleigh, NC, USA). Moreover, the cytotoxicity values of Mex@MLipo and Mex@Lipo (Mex concentrations, 40 µg mL^−1^) to HT-29, HepG-2, and PC-3 cells were also evaluated after co-incubation at 0, 12, and 24 h by the CCK-8 assay.

### 2.11. Mex@MLipo-Cellar Uptake Monitoring and Cell Apoptosis

To investigate the potential impacts of liposome materials on cancer cells and their distributions in cells, real-time monitoring of cancer cells treated with Mex@MLipo or Mex@Lipo was performed. HT-29, HepG-2, and PC-3 cells were seeded in 12-well plates (Corning Co., LTD, Corning, NY, USA) at a density of 2 × 10^5^ cells per well to grow overnight. The fresh media with 40 μL Mex@MLipo and Mex@Lipo labeled with DIO fluorescent dye were replaced to each well at co-incubation time points 0, 3, 6, 9, 12, 15, 18, 21, and 24 h, respectively. Each group had 3 wells in parallel. The supernatant was sucked away uniformly and washed twice with PBS after co-incubation. Then, 4′,6-diamidino-2-phenylindole (DAPI) and nuclear dye (Hoechst 33342) were added to each well and co-incubated with cells for 30 min at 37 °C. Excess dye was cleaned with PBS and was photographed with confocal laser microscope (Ti C2 plus, Nikon Co., LTD, Tokyo, Japan) equipped with a 40× focal oil lens at two excitations wavelengths of 488 nm and 561 nm.

### 2.12. Mechanism of Mex@MLipo Action on Cancer Cells

In order to investigate the relationship between ferroptosis and reactive oxygen species in tumor cells, detection experiment of generation of ROS and change in mitochondrial membrane potential (MMP) were performed in HT-29, HepG-2 and PC-3 cells. Exponentially growing cells were harvested and were plated at the density of 2 × 105 cells per well in 12-well plates (Corning Co., LTD, USA) to grow overnight for ROS and mitochondrial membrane potential detection experiments, respectively. Mex@MLipo or Mex@Lipo materials were added in each well for co-incubation at 0, 6, 9, 12, 15, and 18 h, respectively. Each group had 3 wells in parallel.

### 2.13. Real Time Visualization of ROS Content in Cancer Cells

After co-incubation, the supernatant was cleaned with PBS twice. DCFH-DA (1 mL, 10 μmol L^−1^) (Beyotime, Shanghai, China) diluted with serum-free medium at a ratio of 1:1000 was added to each well and co-incubated with cells for 20 min at 37 °C. Excess probe was washed away with serum-free medium for 3 times and was photographed with laser confocal microscope equipped with a 20× focal oil lens at an excitation wavelength of 488 nm [25]. The fluorescence intensity value was calculated using ImageJ software.

### 2.14. Real Time Visualization of Mitochondrial Membrane Potential Changes

After co-incubation, the supernatant was cleaned with PBS twice. Then, 1 mL of JC-1 Probe (Beyotime, Shanghai, China) diluted with JC-1 buffer solution at a ratio of 1:200 was then added into each well and co-incubated with cells for 20 min at 37 °C. Excess probe was washed away with JC-1 buffer solution for 3 times and was photographed with laser confocal microscope equipped with a 20× focal oil lens at two excitation wavelengths of 514 nm and 585 nm. It is worth noting that JC-1 accumulates in the matrix of mitochondria and forms polymers when the mitochondrial membrane potential is high, which can produce red fluorescence. When the mitochondrial membrane potential is low, JC-1 cannot gather in the matrix of mitochondria. The monomer status of JC-1 can emit green fluorescence [26]. The biggest excitation wavelength of monomer is 514 nm, while that of polymer is 585 nm [27]. The fluorescence intensity values of red and green of the obtained images were processed using ImageJ software (National Institutes of Health, Bethesda, MD, USA). The ratio of the strength of JC-1 polymer to the strength of monomer (red fluorescence intensity/green fluorescence intensity) at different time points was obtained to analyze MMP change trend.

### 2.15. In Vitro Assessment of Glutathione Depletion

Based on Glutathione (GSH), it can react with DTNB to produce a yellow color. DTNB was used as a probe to evaluate the levels of residual GSH in HT29, HepG-2, and PC-3 cells after co-incubation with Mex@MLipo. In brief, HT29, HepG-2, and PC-3 cells were separately seeded in a 24-well plate and cultured for 24 h. Then, Mex@MLipo was added and incubated for 12 and 15 h, respectively. Subsequently, the culture medium was removed, and 500 μL of RIPA lysis buffer was added to each well. DTNB was then added to the collected cell lysate buffer for UV-vis measurement. The change in absorbance was measured at 412 nm to calculate the amount of residual GSH.

### 2.16. Animals and Tumor Models

BALB/c nude male mice were purchased from Shanghai SLAC Laboratory Animal Co., LTD (Shanghai, China). All mice were 6–8 weeks old and kept under specific pathogen free (SPF) conditions in a 12 h light–dark cycle, a room temperature of 20–22 °C, and a humidity of 40–60% for at least 1 week to adapt to the experimental conditions. All animal experiments, animal care, and animal model protocols were approved by the Committee on the Ethics of Animal Experiments of the Institute of Process Engineering at the Southeast University (NO. 20200409006). To establish the subcutaneous xenograft colon tumor model, 8-week-old BALB/c mice were subdermal injected with 5 × 107 HT-29 cells at the right flank.

### 2.17. Biodistribution of Mex@MLipo In Vivo

To explore the targeting and accumulative effect of Mex@MLipo in tumors, HT-29 colon-tumor-bearing mice were randomly assigned to three treatment groups (*n* = 5): Group 1, mice were intravenously injected with DIR/Mex@MLipo with external static magnetic field (SMF) stimulation; Group 2, mice were intravenously injected with DIR/Mex@MLipo; and Group 3, mice were intravenously injected with saline. Then, the mice were scanned by IVIS Spectrum (PerkinElmer, Waltham, MA, USA) in time points (pre injection, 4, 6, 8, 12, 18, and 24 h). Thereafter, the mice were scarified, and main organs (heart, liver, spleen, lung, and kidney) and tumor tissues of mice were collected and imaged using IVIS Spectrum.

### 2.18. In Vivo Antitumor Study

The HT-29 tumor-bearing mice were randomly assigned into two groups (*n* = 5), and each group was injected intravenously with DIR/Mex@MLipo with external SMF stimulation, or DIR/Mex@MLipo twice within 15 days, separately. Meanwhile, the tumor volume and body weight of the mice were recorded every other day for 21 days. After treatment, the mice were sacrificed to collect the tumor and main organ tissues, including the heart, liver, spleen, lung, and kidney. Then, the tumor and main organ tissues were fixed in 4% neutral buffered paraformaldehyde and prepared into paraffin sections for histopathological examination. Firstly, the main organs and tumor sections were cut into slices at a thickness of 10 µm for hematoxylin and eosin (H and E) staining. Furthermore, the tumors sections of 10 µm thickness were prepared and stained with TdT-mediated dUTP Nick-End Labeling (TUNEL). Then, these slices were examined by a bright field TS100/TS100-F optical microscope (Nikon, Tokyo, Japan).

### 2.19. Serum Biochemical Analysis

To analyze the sub-acute toxicity of Mex@MLipo, whole blood samples were separated through centrifugation at 800× *g* for 15 min at a temperature of 4 °C. The resulting samples were used for biochemical analysis. The levels of alanine aminotransferase (ALT), aspartate aminotransferase (AST), alkaline phosphatase (ALP), creatinine, and blood urea nitrogen (BUN) was analyzed using commercial kits Bio Diagnostic Co. (Giza, Egypt) using an autoanalyzer (Cobas INTEGRA 400 plus analyser) (Rayto, Shenzhen, China).

### 2.20. Statistical Analysis

Quantitative data were presented as means ± standard deviation (SD) from sample numbers (*n*). Data from experiments were analyzed using GraphPad Prism 9. Statistical comparisons were made by unpaired Student’s *t*-test (between two groups) and one-way ANOVA (for multiple comparisons). * *p* value < 0.05 was considered statistically significant; ** *p* < 0.01, *** *p* < 0.001, and **** *p* < 0.0001 were extremely significant; NS was considered no significant difference. For quantitative analysis in fluorescence intensity for confocal images, Image J software (National Institutes of Health, Bethesda, MD, USA) was used for densitometric analysis.

## 3. Results

### 3.1. Preparation and Characterization of Mex@MLipo

**Figure 2:**
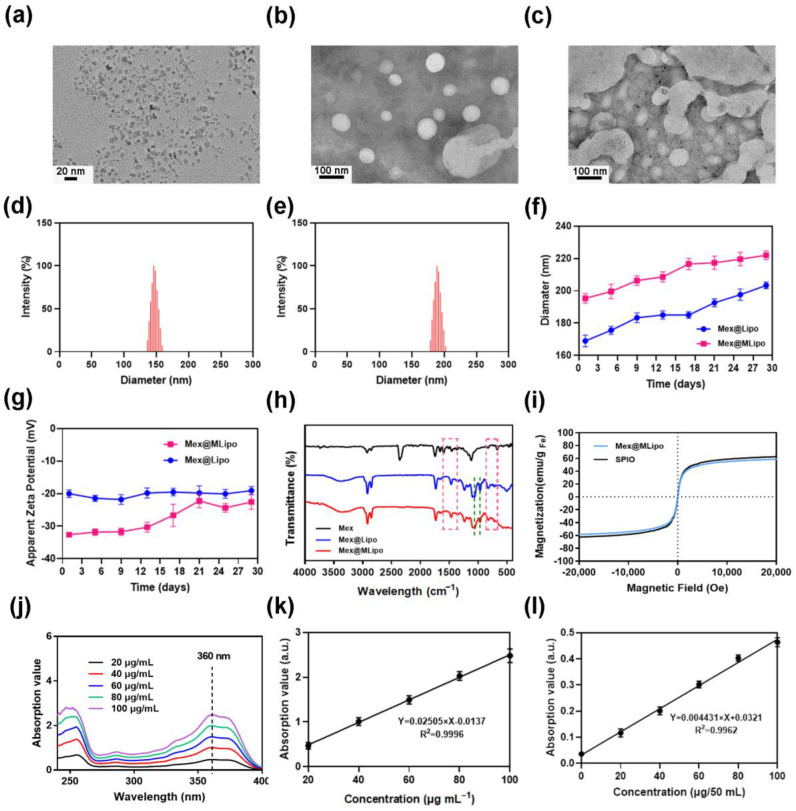
TEM images of (**a**) SPIO, (**b**) Mex@Lipo, and (**c**) Mex@MLipo; Hydrodynamic size distribution of (**d**) Mex@Lipo and (**e**) Mex@MLipo; (**f**) Long-term stability of the liposome diameters and (**g**) Surface Zeta potential measurement in vitro; (**h**) The Fourier transform infrared spectroscopy (FT-IR) spectrum of the Mex, Mex@Lipo, and Mex@MLipo; (**i**) Hysteresis line of SPIO and Mex@MLipo at room temperature (300 K); (**j**) The ultraviolet (UV) spectrogram of Mex–methanol solution; (**k**) Standard curve of Mex concentration measured by UV spectrophotometer; (**l**) Standard curve of iron concentration measured by UV spectrophotometer.

The liposomes loaded with Mex within the lipid bilayer (Mex@Lipo) and γ-Fe_2_O_3_ SPIO encapsulated in the core (Mex@MLipo) were prepared by hydration and a membrane-extrusive method. TEM image showed that the diameter of SPIO ranged between 8 and 10 nm (Figure 2a), and the morphology of Mex@Lipo was overall uniform, showing a smooth surface and visible edges (Figure 2b). Mex@MLipo had SPIO trapped and dispersed in the core, indicated by a visible high electron density area (Figure 2c). The Mex@Lipo liposomes had a size of 143 nm and a polydispersity index (PDI) of 0.11 (Figure 2d). The Mex@MLipo had an average size of 176 nm and a PDI of 0.16 (Figure 2e), indicating that the addition of hydrophilic SPIO in the core of the liposomes caused a size increment. The Mex@Lipo and Mex@MLipo had a Zeta potential of −32.44 mV and −20.04 mV, respectively. The stability of Mex@Lipo and Mex@MLipo was verified by continuous measurement of particle size and surface potential for 30 days. Results showed that no significant changes were observed (Figure 2f,g). 

To confirm the presence of Mex in liposomes, the purified liposomes were lyophilized into powder, scanned by FT-IR, and analyzed by comparison with Mex powder as a control group. The absorption peaks at 860–670 cm^−1^ and 1610–1370 cm^−1^ corresponded to the vibrational frequencies of benzene ring=CH bending and aromatic ring C=C stretching, respectively. It was consistent with the benzene ring structure of free Mex and appeared in the Mex@Lipo and Mex@MLipo. Unique signals at 990–950 cm^−1^ of P-O-H stretching vibration in HPO_4_^2−^ and 1070 cm^−1^ of PO_4_^3−^ symmetric stretching vibrations in HPO_4_^2−^, respectively, indicating the presence of phospholipid molecules (Figure 2h and Figure S2). These results demonstrated that the Mex molecules were successfully combined with the lipid bilayers of the Mex@Lipo and Mex@MLipo formulations.

Figure 2i showed that Mex@MLipo exhibited good superparamagnetism at 300 K with a saturation magnetization intensity of 58.55 emu/g Fe, which was comparable to the saturation magnetization intensity of SPIO. It indicated that Mex@MLipo successfully encapsulated SPIO without affecting its magnetic properties, performed good magnetic response, and could be magnetized and enriched under the conditions of an applied static magnetic field.

UV-vis spectroscopy was used to further measure the encapsulation concentration of Mex in the liposomes. An evident linear relationship was observed between the absorbance and Mex concentration. Result in Figure 2j showed that the maximum Mex absorbance was 360 nm for different Mex concentrations (20–100 μg mL^−1^). The standard curve of Mex was calculated and shown in Figure 2k. The standard curve and the UV-vis spectra of the sample revealed that the encapsulation efficiency of Mex in the liposomes was 65.86% with a concentration of 1094.68 μg mL^−1^. The concentration of encapsulated SPIO in the liposomes was measured to be 461.2 μg mL^−1^ using a standard iron concentration curve (Figure 2l). The encapsulation efficiency of SPIO in Mex@MLipo was determined to be 76.86%.

### 3.2. In Vitro Evaluation of Toxicity of Mex@MLipo to Different Tumor Cells

**Figure 3:**
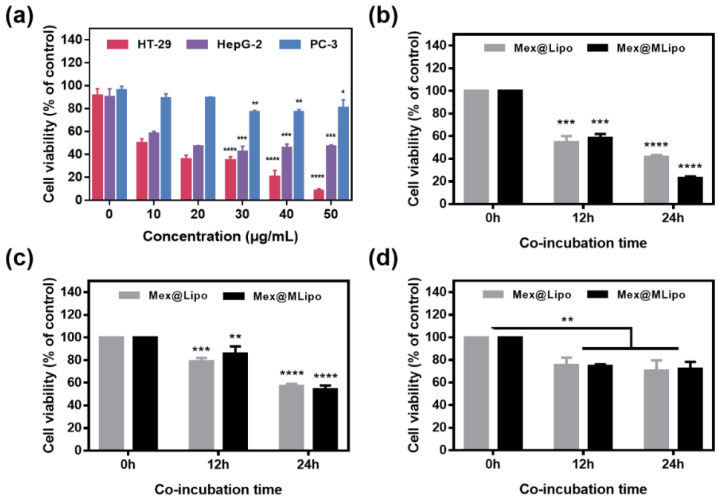
(**a**) Cell Viability of HT-29, HepG2, and PC-3 cells when incubated with Mex@MLipo at 24 h under different concentrations of Mex (0, 10, 20, 30, 40, and 50 µg mL^−1^). Cell viability of (**b**) HT-29, (**c**) HepG-2, and (**d**) PC-3 treated with Mex@Lipo and Mex@MLipo under 40 µg mL^−1^ concentration of Mex. Error bars: mean ± SD (*n* = 3). The statistical significance is indicated by **** *p* < 0.0001, 0.0001 < *** *p* ≤ 0.001, 0.001 < ** *p* ≤ 0.01, and 0.01 < * *p* ≤ 0.05, in comparison between control group and experimental group using an unpaired Student’s *t*-test (two-tailed).

To evaluate the therapeutic function of Mex@MLipo in cancer therapy and determine the optimal concentration with maximal antitumor activity and minimal toxicity, three types of cancer cells (HT-29, HepG-2, and PC-3) were treated with varying concentrations of Mex (0–50 µg mL^−1^) for 24 h in vitro. The antitumor activity of Mex was evaluated by measuring cell viability using the CCK-8 proliferation assay. At concentration of 40 μg mL^−1^, Mex showed significant antitumor activity against HT-29 cells, with a cell viability of 20.16%. HepG-2 cells showed relatively low antitumor activity at this concentration, with a cell viability of 47.08%. When the concentration was increased to 50 µg mL^−1^, Mex did not result in lower cell viability in HepG-2 cells. However, Mex did not exhibit significant toxicity against PC-3 cells in the concentration range of 0–50 µg mL^−1^, and its lowest cell viability was 76.66% (Figure 3a). Based on these results, a concentration of 40 µg mL^−1^ of Mex was selected for subsequent experiments. The cytotoxicity Mex@MLipo was then evaluated against HT-29, HepG-2, and PC-3 cells at 0, 12, and 24 h after co-incubation using the CCK-8 proliferation assay. The results showed that both Mex@MLipo and Mex@Lipo exhibited strong cytotoxicity, and Mex@MLipo showed more cytotoxicity (cell viability was close to 23.11% at co-cultured 24 h) than Mex@Lipo (cell viability was 41.55% at co-cultured 24 h), for the HT-29 cells, whereas Mex@MLipo co-cultured at 12 h showed less cytotoxicity than Mex@Lipo (Figure 3b). It suggested that the presence of SPIO delayed the toxicity of liposomal materials but could ultimately serve to enhance the toxicity of liposomal materials. For HepG-2 cells, both Mex@MLipo and Mex@Lipo exhibited similar and lower cell cytotoxicity (approximately 55% cell viability at co-cultured 24 h) (Figure 3c), indicating that only Mex acted on HepG-2 cells, and the presence of SPIO did not cause additional cytotoxicity. Mex@MLipo and Mex@Lipo showed low cytotoxicity to PC-3 cells (cell viability was close to 75% at co-cultured 12 and 24 h) (Figure 3d). It suggested that Mex has non-evident cytotoxicity on PC-3 cells, and SPIO had no significant impact on its cytotoxicity.

### 3.3. Cellular Uptake of Mex@MLipo

To confirm whether Mex can enter the nucleus to exert its antitumor effects better when encapsulated in a liposomal carrier, tumor cells were co-cultured with DIO fluorescently labeled Mex@MLipo or Mex@Lipo and imaged at different co-culture times (0, 3, 6, 9, 12, 15, 18, 21, 24 h) using laser confocal microscopy. 

**Figure 4:**
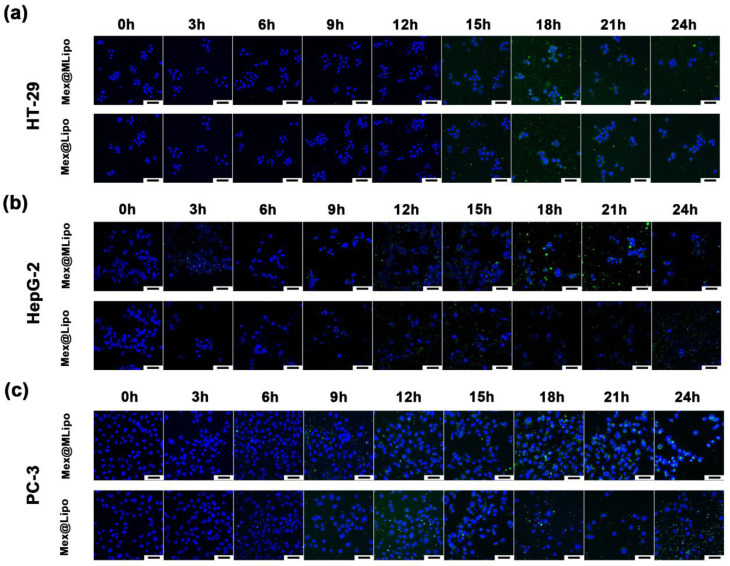
Confocal images of (**a**) HT-29 cell, (**b**) HepG-2 cell, and (**c**) PC-3 cell co-cultured with DIO/Mex@MLipo and DIO/Mex@Lipo at 0, 3, 6, 9, 12,15, 18, 21, and 24 h. Blue fluorescence represents nucleus and green fluorescence represents liposome materials. All scale bars: 50 μm.

After 12 h of co-culture, no visible Mex@MLipo was found in the vicinity of HT-29 cells, and the cells remained in a normal state. Mex@MLipo was around the nucleus after 15 h incubation, with an increase in number observed from 18 to 21 h. However, the number of cells in the visual field decreased gradually from 21 to 24 h, indicating cell apoptosis. In comparison, the condition of HT-29 cells treated with Mex@Lipo was similar to that of Mex@MLipo during the first 18 h of treatment. However, the number of cells in the visual field decreased within 18 h treatment in the Mex@Lipo group, indicating an earlier onset of cell apoptosis compared to the Mex@MLipo group (Figure 4a and Figure S3). This observed delay in the function of Mex in HT-29 cells treated with Mex@MLipo was thought to be related to steric hindrance caused by the presence of SPIO. However, this temporary hindrance did not affect the toxicity of Mex@MLipo on HT-29 cells. In fact, a significant decrease in the number of cells in the visual field was observed at both 21 and 24 h of co-incubation, with the cell state weaker in the Mex@MLipo group compared to the Mex@Lipo group. These findings suggested that Mex@MLipo could exert even stronger toxicity and SPIO facilitate the materials to enter the nuclei under prolonged treatment.

The cellular uptake of Mex@MLipo by HepG-2 cells was similar to that of Mex@Lipo within the first 12 h treatment period. During the initial 6 h treatment period, no material appeared around the cells. From 9 to 12 h, material gradually accumulated around the cells. In the Mex@MLipo group, an increasing number of fluorescent signals was observed to cluster in the nucleus during the 15–21 h treatment period. From 21 to 24 h, a substantial decrease in the number of cells was observed. In the Mex@Lipo group, from 15 h onwards, there was a dramatic decrease in the number of cells with observed fragmented nucleus structures (Figure 4b and Figure S4). The use of liposomes as carriers facilitated the intracellular uptake of Mex in HepG-2 cells, allowing Mex to more easily enter the nucleus and exert its antitumor effects. However, the inclusion of SPIO did not show additional functions, which was consistent with the results of cell toxicity experiments. These findings suggested that liposomes could serve as efficient carriers for the delivery of Mex to HepG-2 cells, yet the inclusion of SPIO showed no additional benefits. Further investigations are needed to illustrate the underlying mechanisms and optimize the design of such drug delivery systems for clinical applications.

In the co-culture of PC-3 cells with DIO/Mex@MLipo or DIO/Mex@Lipo, there was a limited reduction in cell numbers and apoptosis, indicating that these materials did not exhibit significant cytotoxicity against the cells. The observed accumulation of the materials around the cells after 9 h treatment further supported the notion of their low cytotoxicity. At 12 h incubation, the materials were bound to the cells and formed green fluorescent dots. The fluorescence remained in the nucleus for up to 24 h. Apart from the presence of these dots, the cells exhibited normal morphological activity and remained viable (Figure 4c and Figure S5).

To summarize, the results showed that Mex@MLipo and Mex@Lipo exhibited evident cellular uptake in HT-29, HepG-2, and PC-3 cells. Notably, the fluorescence signals observed in cells treated with Mex@MLipo after 15 h incubation were significantly stronger than those in cells treated with Mex@Lipo, indicating that the presence of SPIO facilitated the intracellular internalization of the liposomal drug delivery system. In HT-29 cells, the presence of SPIO had a dual effect on Mex action on the tumor. On one hand, it prolonged the duration of the drug’s action. On the other hand, with the increase in incubation time, SPIO induced apoptosis in the cells. In contrast, SPIO did not induce apoptosis in HepG-2 and PC-3 cells. Instead, they caused further tight binding of the material to the nucleus, which might protect the survival of tumor cells to some extent. These findings suggest that the inclusion of SPIO in liposomal drug delivery systems can enhance their cellular uptake and potentially modulate their therapeutic effects on different types of cancer cells. Further studies are warranted to elucidate the underlying mechanisms and optimize the design of such systems for clinical applications.

### 3.4. Evaluation of the Mechanism of Enhanced Antitumor Capacity of Mex@MLipo

**Figure 5:**
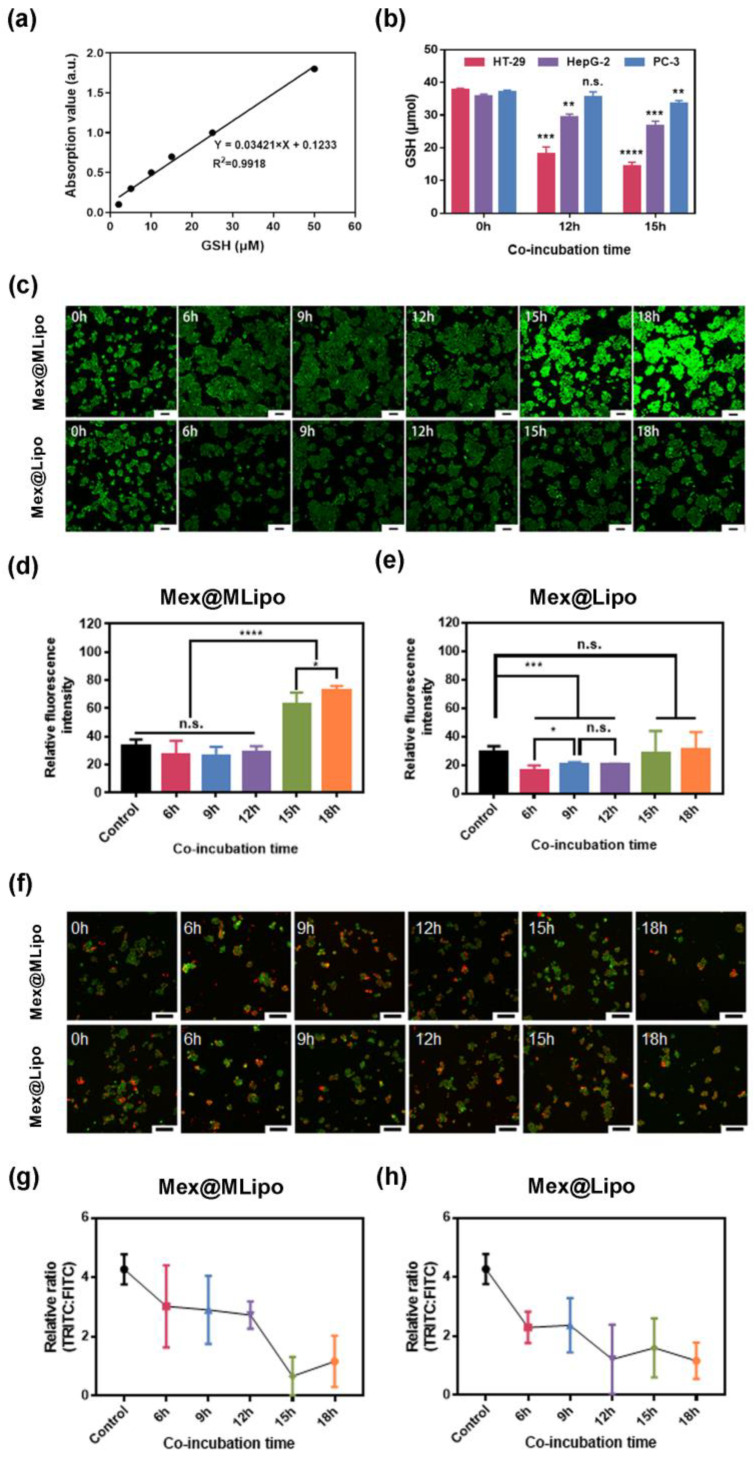
(**a**) Standard curve of GSH concentration measured by ELIASA. (**b**) Concentrations of intracellular GSH of HT-29, HepG-2, and PC-3 cells after co-incubation with Mex@MLipo for 0, 12, and 15 h. (**c**) Images of intracellular ROS fluorescence of HT-29 cells after co-incubation with Mex@MLipo and Mex@Lipo. Green fluorescent: intracellular ROS levels. Scale bars: 100 μm. Quantitative analysis of fluorescence intensity for (**d**) Mex@MLipo and (**e**) Mex@Lipo; (**f**) Images of JC-1 fluorescence of HT-29 cells after co-incubation with Mex@MLipo and Mex@Lipo and (**g**,**h**) quantitative analysis of fluorescence intensity, respectively. Red fluorescent: JC-1 in polymer form; Green fluorescent: JC-1 in monomeric form. Scale bars: 100 μm. Error bars: mean ± SD (*n* = 5). The statistical significance is indicated by **** *p* < 0.0001, 0.0001 < *** *p* ≤ 0.001, 0.001 < ** *p* ≤ 0.01, and 0.01 < * *p* ≤ 0.05; n.s.: Not Statistically Significant. using an unpaired Student’s *t*-test (two-tailed).

Next, considering that magnetic nanoparticles have the potential to induce iron death in tumor cells, we analyzed the changes in intracellular GSH concentration after treatment with Mex@MLipo in different types of tumor cells using a GSH assay kit to investigate whether the activity of different types of tumor cells was related to iron death induced by magnetic nanoparticles. Based on the GSH standard concentration curves (Figure 5a), we observed that Mex@MLipo significantly reduced the concentration of GSH in HT-29 and HepG-2 cells in a time-dependent manner. In contrast to these two cells, Mex@MLipo had a weaker effect on the concentration of GSH within PC-3 cells (Figure 5b). The consistency of the inhibition of GSH concentration with the previous results on cell viability suggested that Mex@MLipo may have been affecting the redox environment in tumor cells through ferroptosis, thereby affecting tumor activity.

To further investigate whether the killing effect of Mex@MLipo on tumor cells was achieved by inducing ferroptosis in tumor cells, we further explored the changes in intracellular ROS levels and mitochondrial membrane potentials in HT-29, HepG-2, and PC-3 cells after treatment with Mex@Lipo or Mex@MLipo for different time periods. As shown in Figure 5c, a significant increase in intracellular ROS levels could be clearly observed in HT-29 cells co-incubated with Mex@MLipo for 15 h (Figure 5c,d). However, ROS levels within HT-29 cells co-cultured with Mex@Lipo showed no evident change with extended co-incubation time (Figure 5c,e), suggesting that SPIO uptake by the cells could significantly enhance the production of ROS within HT-29 cells. Furthermore, a significant decrease in mitochondrial membrane potential was observed in the HT-29 cells after 12 h of co-incubation with Mex@MLipo compared to HT-29 cells co-incubated with Mex@Lipo (Figure 5f,g). The results of these experiments indicated that Mex@MLipo endocytosed by cells induced ferroptosis in HT-29 cells after the depletion of intracellular GSH, followed by a dramatic change in the intracellular redox environment, leading to a killing effect on tumor cells.

In addition, we investigated the effects of Mex@MLipo and Mex@MLipo on HepG-2 and PC-3 cells, respectively. The results showed that the generation and decrease in ROS and mitochondrial membrane potential were similar on HepG-2 cells after co-incubation with Mex@MLipo or Mex@MLipo separately, indicating that SPIO did not induce ferroptosis or cause significant cytotoxicity to HepG-2 cells (Figure S6). However, the Mex@MLipo produced more ROS intracellularly and caused a greater decrease in mitochondrial membrane potential compared to Mex@Lipo, leading to apoptosis after 9 h of treatment. This suggested that a higher proportion of Mex loaded in liposomes could prolong the duration of action time on tumor cells in the presence of no SPIO. However, there were no significant changes in ROS, mitochondrial membrane potential, or apoptosis signal in PC-3 cells after co-culture with Mex@MLipo or Mex@Lipo, respectively, indicating that neither Mex nor SPIO were effective against PC-3 cells (Figure S7). These findings were consistent with the cytotoxicity and endocytosis experiments presented previously, suggesting that Mex@MLipo and Mex@MLipo were not effective against PC-3 cells.

Collectively, in the results of the experiments on GSH and ROS concentrations, mitochondrial membrane potentials in different cell types showed that the same dose of SPIO exerted different effects in different cells. In HT-29 cells, SPIO produced more reactive oxygen species to induce ferroptosis and promoted apoptosis together with Mex. In contrast, in HepG-2 and PC-3 cells, SPIO did not produce more reactive oxygen species, and excess SPIO was not effective in killing tumor cells.

### 3.5. Biodistribution of Mex@MLipo

**Figure 6:**
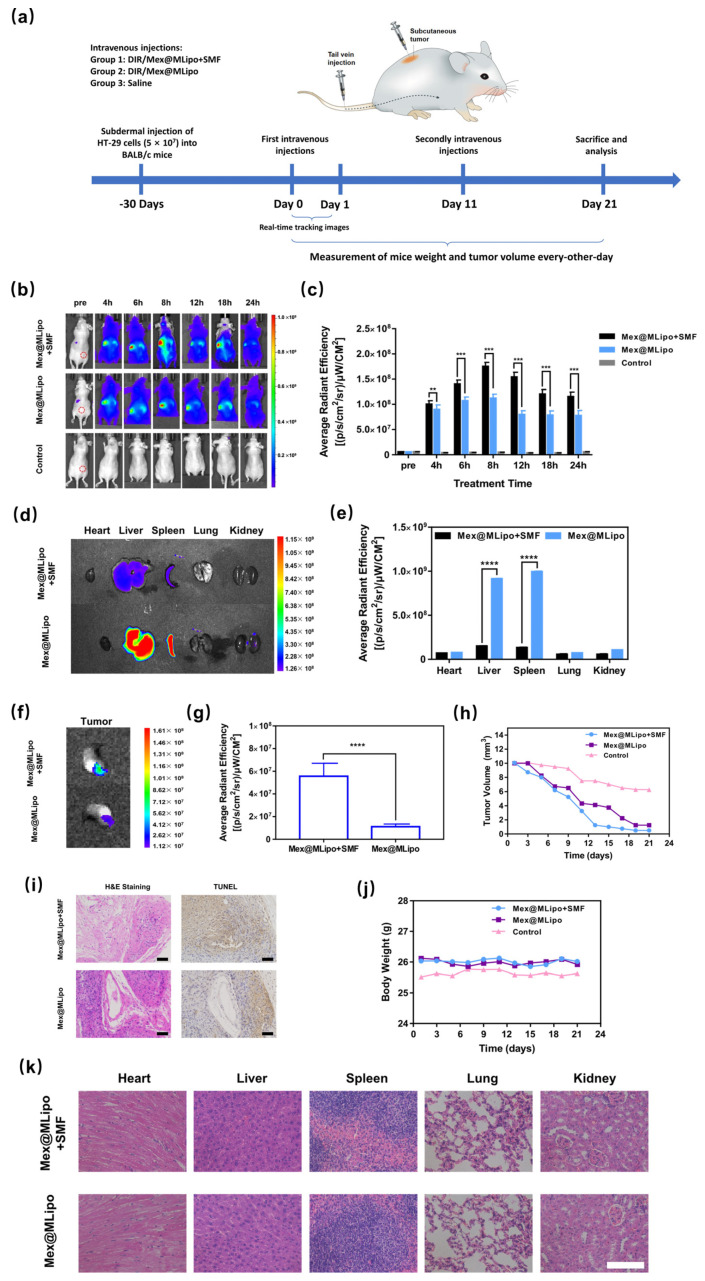
(**a**) Experimental flow timeline; (**b**) Plot of NIR fluorescence imaging over time (pre, 4, 6, 8, 12, 18, 24 h) in Mex@MLipo+SMF group, Mex@MLipo group, and blank control group mice; (**c**) Histogram of NIR fluorescence signal intensity over time at the tumor site (ROI) in Mex@MLipo+SMF group, Mex@MLipo group, and blank control group mice; (**d**) NIR fluorescence imaging maps of isolated organs (heart, liver, spleen, lung, and kidney) after injection of Mex@MLipo with adjuvant SMF stimulation and after injection of Mex@MLipo alone; (**e**) Histograms for quantitative analysis of NIR fluorescence signal intensity of isolated organs; (**f**) NIR fluorescence imaging maps of tumor issues after injection of Mex@MLipo with adjuvant SMF stimulation and after injection of Mex@MLipo alone; (**g**) Histograms for quantitative analysis of NIR fluorescence signal intensity of isolated tumors; (**h**) Folding plots of tumor volume change in Mex@MLipo+SMF group, Mex@MLipo group, and blank control nude mice; (**i**) Pictures of H and E staining and TUNEL apoptosis staining of tumor tissue sections from Mex@MLipo+SMF group and Mex@MLipo group mice, scale bar: 50 μm; (**j**) Folding plots of body weight change in Mex@MLipo+SMF group, Mex@MLipo group and blank control nude mice; (**k**) H and E stained images of heart, liver, spleen, lung, and kidney tissue sections of mice in Mex@MLipo+SMF group and Mex@MLipo group, the scale bar in the figure is 50 μm. Note: Day 0 in plot is the day of the first tail vein injection, which is day 30 on the time axis in (**a**). Error bars: mean ± SD (*n* = 5). The statistical significance is indicated by **** *p* < 0.0001, 0.0001 < *** *p* ≤ 0.001, 0.001 < ** *p* ≤ 0.01, using an unpaired Student’s *t*-test (two-tailed).

As magnetic nanoparticles have excellent responsiveness to external magnetic fields, in order to investigate the tumor targeting ability of Mex@MLipo in vivo, BALB/c nude mice were used as the animal model to evaluate the distribution of Mex@MLipo in vivo with and without a static magnetic field (SMF), using a near-infrared (NIR) fluorescence in vivo imaging system (Figure 6a).

Firstly, to study the enhanced accumulation effect of Mex@MLipo in tumor regions by applying SMF in vitro, Mex@MLipo labeled with DIR was administered into tumor-bearing nude mice via the tail vein. The distribution and accumulation of DIR/Mex@MLipo in the nude mice were photographed by NIR imaging of the nude mice. The results of in vivo NIR imaging are shown in Figure 6b. The tumor regions of nude mice showed evident fluorescence signals, and signals were overall stable from 4 to 24 h and reached a peak around 8 h. The fluorescence signal in the tumor region of the Mex@MLipo+SMF group was stronger than in the Mex@MLipo group, indicating that the Mex@MLipo could accumulate more at the tumor site under the effect of the applied SMF. The average fluorescence intensity at the tumor site was analyzed using Living Image software (Nikon, Tokyo, Japan), and the results are shown in Figure 6c. The fluorescence intensity at the tumor area peaked after 8 h of tail vein injection and remained relatively stable for 24 h. 

To further investigate the distribution of Mex@MLipo in the major organs and tumor tissues of mice after the application of SMF, NIR imaging was performed on the hearts, livers, spleens, lungs, kidneys, and tumors of anatomically fixed mice. The average fluorescence intensities of organs and tumor tissues were analyzed graphically. As shown in Figure 6d, the fluorescence intensities of the livers and spleens of mice without SMF induction was extremely high, probably because DiR/Mex@MLipo was heavily trapped in the livers and spleens. The quantitative analysis of fluorescence intensity showed that DIR/Mex@MLipo did not accumulate significantly in the heart, lung, and kidney; the fluorescence intensity of the liver tissue in the Mex@MLipo group was 6.13 times higher than that in the Mex@MLipo+SMF group; and the fluorescence intensity of spleen tissue in the Mex@MLipo group was 7.53 times higher than that in the Mex@MLipo+SMF group (Figure 6e). The fluorescence intensity of tumor tissues was significantly lower than that of tumor tissues with SMF applied (Figure 6f), and the fluorescence intensity of Mex@MLipo + SMF group was 4.83 higher than that in Mex@MLipo group (Figure 6g). These results indicated that the addition of SMF induction at the tumor sites in mice could cause Mex@MLipo to reduce the sequestration of the liver and spleen reticuloendothelial systems and accumulate more at the tumor regions.

### 3.6. In Vivo Antitumor Therapy of Mex@MLipo

To investigate the antitumor ability of SMF-assisted Mex@MLipo, HT-29 tumor-bearing mice were used as animal models. Mice were randomly divided into three groups and intravenously injected with Mex@MLipo with adjuvant SMF guidance, Mex@MLipo, and saline on day 0 and day 10, respectively (Figure 6a). At the same time, the tumor volume and weight of the mice were recorded every other day during the experimental period. The changes in tumor volume in mice are shown in Figure 6h. The tumor volume of mice in the blank control group gradually decreased, indicating that the mice themselves had some self-healing ability for subcutaneous tumors, but the decreasing trend was significantly weaker than those of the two experimental groups. In addition to the effect of the self-healing ability of the mice themselves, the tumor volume of mice in the Mex@MLipo+SMF group decreased significantly compared with those in the Mex@MLipo group and the Mex@MLipo group, and the tumor volume of mice in the Mex@MLipo+SMF group decreased more significantly than those in the Mex@MLipo group after the enrichment targeting effect of the applied static magnetic field. This indicated that the combination treatment of Mex@MLipo and SMF could enhance the tumor treatment effect by accumulating more materials in the tumor lesion site under the targeting effect of static magnetic field.

To further verify the in vivo antitumor performance of Mex@MLipo, the tumor tissues of the treated mice were dissected into paraffin sections and subjected to H and E staining and TUNEL staining, and the results were observed under a light microscope, as shown in Figure 6i. TUNEL staining showed that the tumor tissues of both Mex@MLipo+SMF- and Mex@MLipo-treated mice were cavernous, with discontinuous distributions of tumor tissues and large areas of necrosis, sparse cytoplasm, blurred cell boundaries, and finely granular nuclei, indicating that the tumor killing ability of Mex@MLipo was more prominent under the in vitro assistance of a static magnetic field, and the treatment effect was significantly better than that of the pure liposome treatment group.

To verify the bio-safety of the treatment method, the body weights of mice in the treatment cycle were measured, and the changes in body weights of mice are shown in Figure 6j. The body weight of mice did not show significant changes after receiving Mex@MLipo and SMF treatment and was maintained within the normal range. It thus indicated that the treatment regimen did not affect the normal physiological activities of mice.

The hearts, livers, spleens, lungs, and kidneys of the mice were dissected, and paraffin tissue sections of the organs were made for H and E staining to further assess the biosafety. The staining results of the sections are shown in Figure 6k. It can be observed that the tissue structure of the main organs of the mice is intact, the nuclei and cytoplasm are intact and clear, and no histological lesions are observed, suggesting that the organ tissues of the mice did not show toxic side effects after receiving Mex@MLipo + SMF and Mex@MLipo treatment. In addition, the serum biochemical assays of mice injected intravenously with Mex@MLipo for 10 days showed that all the tested indexes were within the normal range (Figure S8). Collectively, the above results demonstrated the good biosafety of the Mex@MLipo treatment in the experiment.

## 4. Discussion

In this study, liposomes loaded with both SPIO and Mex (Mex@MLipo) were fabricated. The morphological characterization showed that Mex@MLipo had a uniform diameter and was stable in a time range of 30 days. We successfully encapsulated SPIO and Mex within the nano-liposomes with a high encapsulation rate. We investigated the diffusions and toxicities of Mex@MLipo materials on three different types of tumor cells and studied the mechanisms of action of Mex and SPIO on tumor cells. Our results showed that the steric hindrance of SPIO delayed the action of Mex on HT-29 tumors but did not impede the ability of Mex and SPIO to kill tumor cells. Instead, the presence of SPIO lifted the level of intracellular oxidative stress, induced ferroptosis, and increased the strength of cell death. As a result, the toxicity produced by Mex@MLipo was more evident than that produced by Mex@Lipo on HT-29 cells. No ferroptosis was observed in HepG-2 and PC-3 cells, and SPIO only enhanced the connection between liposomes and the nucleus. Although Mex@MLipo did not have a prominent therapeutic effect on PC-3 cells, the SPIO used in the experiment had strong adhesion to the nucleus and high stability. Thus, the incorporation of SPIO may be useful for subsequent real-time angiography and imaging.

Mex@MLipo had a prominent passive targeting ability upon tumors under the effect of static magnetic field induction, with significant accumulation in the tumor lesion area. After treatments by tail vein injection on day 1 and 10 in the tumor treatment cycle, mice treated with Mex@MLipo+SMF showed a significant reduction in tumor volume compared to the pure Mex@MLipo treatment group, which evidently outperformed the blank control group. The distribution of Mex@MLipo in major organs and tumor tissues was analyzed. The high enrichment in tumor focal tissues improved the efficacy and biosafety by reducing the sequestration of Mex@MLipo by the reticuloendothelial system in the presence of applied static magnetic field induction, thereby reducing its accumulation in the liver and spleen. After mice received Mex@MLipo+SMF and pure Mex@MLipo treatment, the major organ tissues were structurally intact with clear margins, and the mice had normal serum liver and kidney function indices, indicating that Mex@MLipo had good biosafety. After the mice received Mex@MLipo+SMF and pure Mex@MLipo treatment, there was no significant trend in change in body weight compared with the control group, which showed good biometabolic ability, further supporting the reliable biosafety of the treatment method. 

In conclusion, this therapeutic combination strategy through a combination of chemotherapeutic agents and magnetic nanoparticles loaded with liposomes provides an effective and safe synergy for the treatment of colorectal adenocarcinoma in future clinical applications.

## Data Availability

The data underlying this article are available in the article and in its online Supplementary Material.

## Supplementary Materials

The following supporting information can be downloaded at: https://www.mdpi.com/article/10.3390/pharmaceutics15072012/s1, Figure S1: TEM images and hydrodynamic size distribution of DIO/Mex@Lipo and DIO/Mex@MLipo; Figure S2: The Fourier transform infrared spectroscopy (FT-IR) spectrum of the Mex, Mex@Lipo, and Mex@MLipo; Figure S3: Confocal images of HT-29 cell co-cultured with DIO/Mex@MLipo and DIO/Mex@Lipo at 0, 3, 6, 9, 12,15, 18, 21, and 24 h; Figure S4: Confocal images of HepG-2 cell co-cultured with DIO/Mex@MLipo and DIO/Mex@Lipo at 0, 3, 6, 9, 12,15, 18, 21, and 24 h; Figure S5: Confocal images of PC-3 cell co-cultured with DIO/Mex@MLipo and DIO/Mex@Lipo at 0, 3, 6, 9, 12, 15, 18, 21, and 24 h; Figure S6: Evaluation of the mechanism of enhanced antitumor capacity of Mex@MLipo for HepG-2 cells; Figure S7: Evaluation of the mechanism of enhanced antitumor capacity of Mex@MLipo for PC-3 cells; Figure S8: Serum biochemical analysis in the different treatment groups.
