# Supplementary material for: A Moexitecan Magnetic Liposomal Strategy for Ferroptosis-Enhanced Chemotherapy
Source: Pharmaceutics. 2023 Jul 24;15(7):2012. doi: 10.3390/pharmaceutics15072012 (PMC10386037; doi:10.3390/pharmaceutics15072012)
Supplement: Supplementary file 1 [file pharmaceutics-15-02012-s001.zip › pharmaceutics-2457009-supplementary.pdf]

pharmaceutics

MDPI

# Supplementary Information

# A Moexitecan Magnetic Liposomal Strategy for Ferroptosis-Enhanced Chemotherapy

**Weiling Miao †, Yang Liu †, Jian Tang, Tiandong Chen and Fang Yang ***

State Key Laboratory of Digital Medical Engineering, Jiangsu Key Laboratory for Biomaterials and Devices, School of Biological Sciences and Medical Engineering, Southeast University; Nanjing 210096, China; weilling_miao@seu.edu.cn (W.M.); lyseubme@seu.edu.cn (Y.L.); jiantang@seu.edu.cn (J.T.); 230218251@seu.edu.cn (T.C.)

* Correspondence: yangfang2080@seu.edu.cn

† These authors contributed equally to this work.

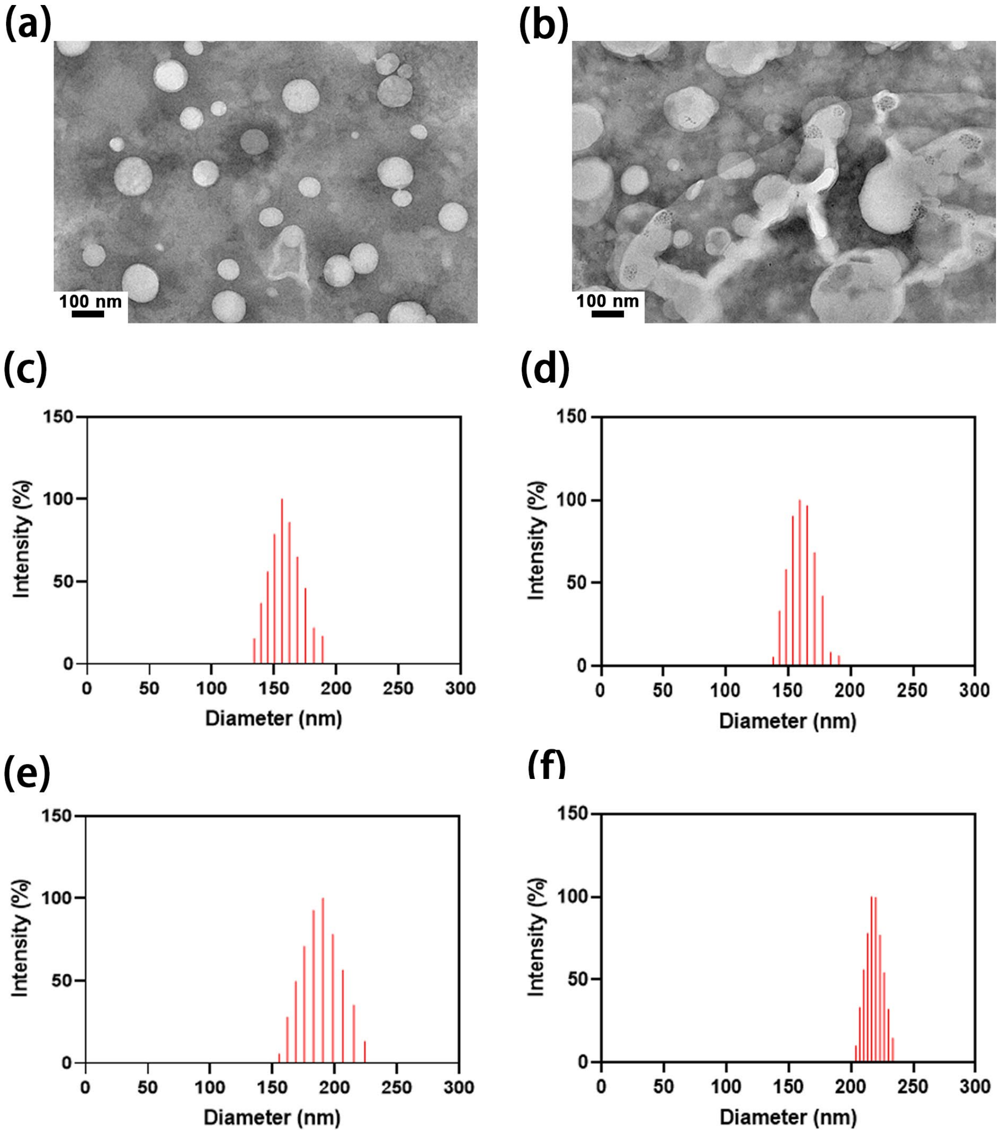


**Figure S1.** TEM images of (**a**) DIO/Mex@Lipo and (**b**) DIO/Mex@MLipo; Hydrodynamic size distribution of (**c**,**d**) Mex@Lipo and (**e**,**f**) Mex@MLipo.

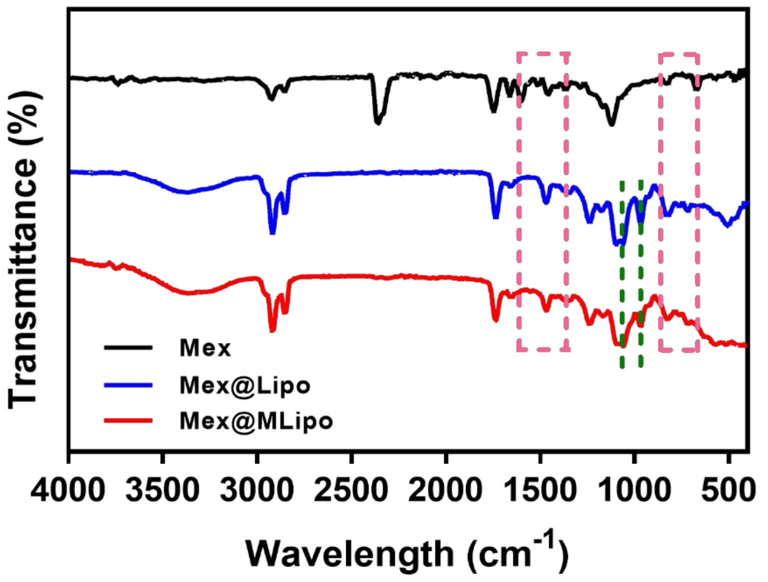


**Figure S2.** The Fourier transform infrared spectroscopy (FT-IR) spectrum of the Mex, Mex@Lipo and Mex@MLipo.

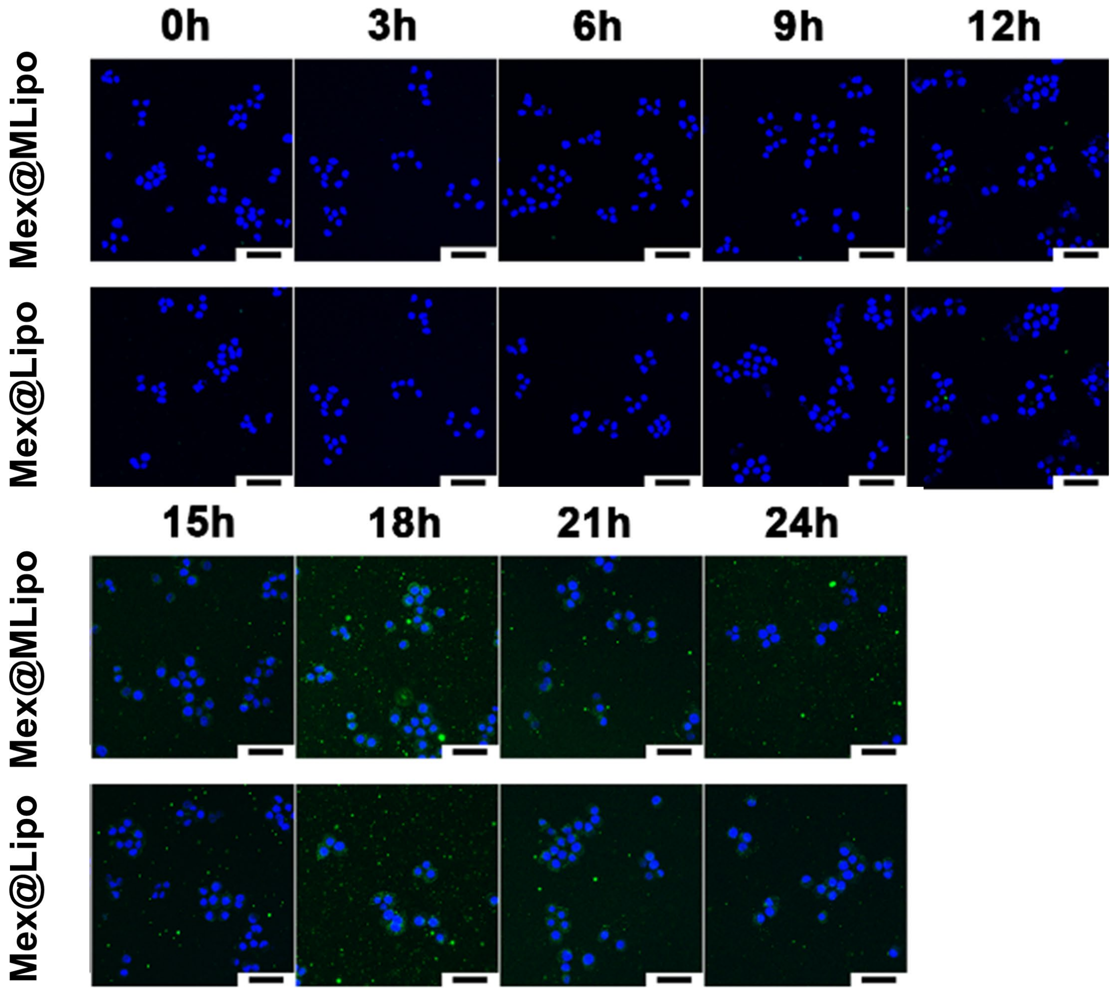


**Figure S3.** Confocal images of HT-29 cells co-cultured with DIO/Mex@MLipo and DIO/Mex@Lipo at 0, 3, 6, 9, 12,15, 18, 21, and 24 h. Blue fluorescence represents nucleus and green fluorescence represents liposome materials. All scale bars: 50 μm.

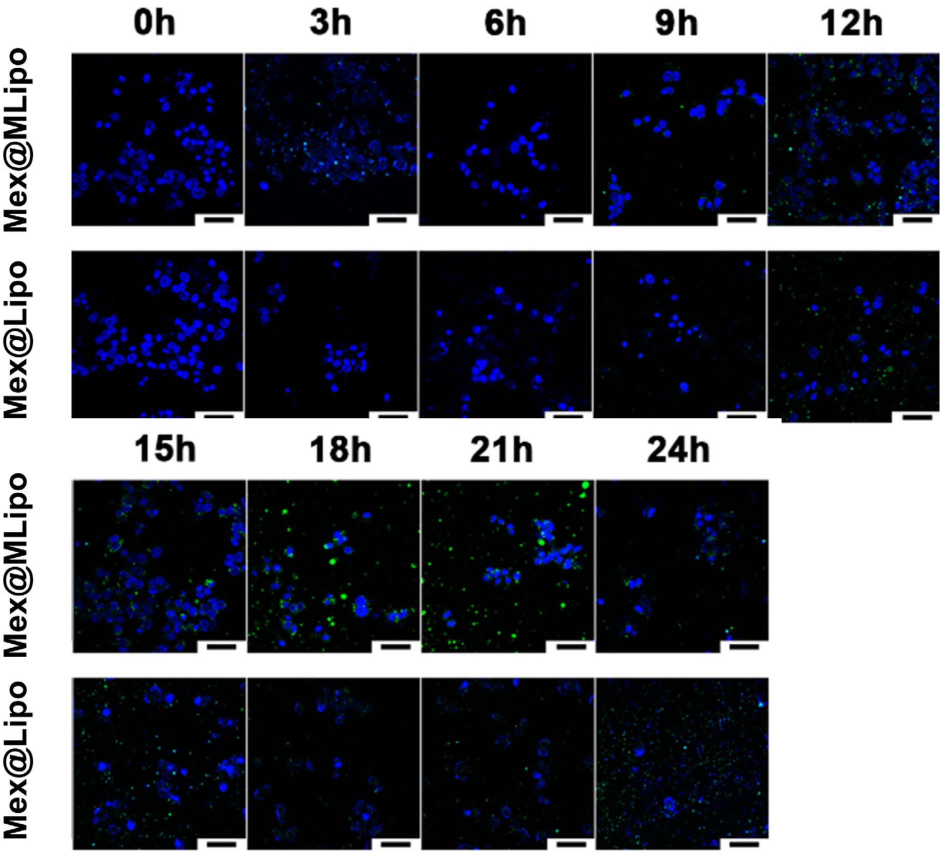


**Figure S4.** Confocal images of HepG-2 cells co-cultured with DIO/Mex@MLipo and DIO/Mex@Lipo at 0, 3, 6, 9, 12,15, 18, 21, and 24 h. Blue fluorescence represents nucleus and green fluorescence represents liposome materials. All scale bars: 50 μm.

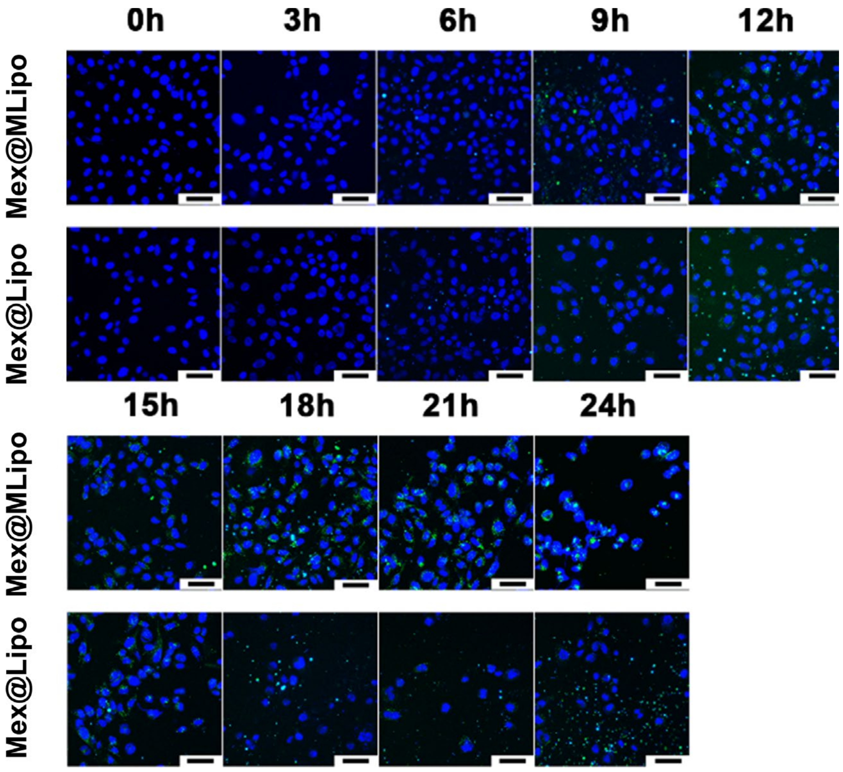


**Figure S5.** Confocal images of PC-3 cells co-cultured with DIO/Mex@MLipo and DIO/Mex@Lipo at 0, 3, 6, 9, 12,15, 18, 21, and 24 h. Blue fluorescence represents nucleus and green fluorescence represents liposome materials. All scale bars: 50 μm.

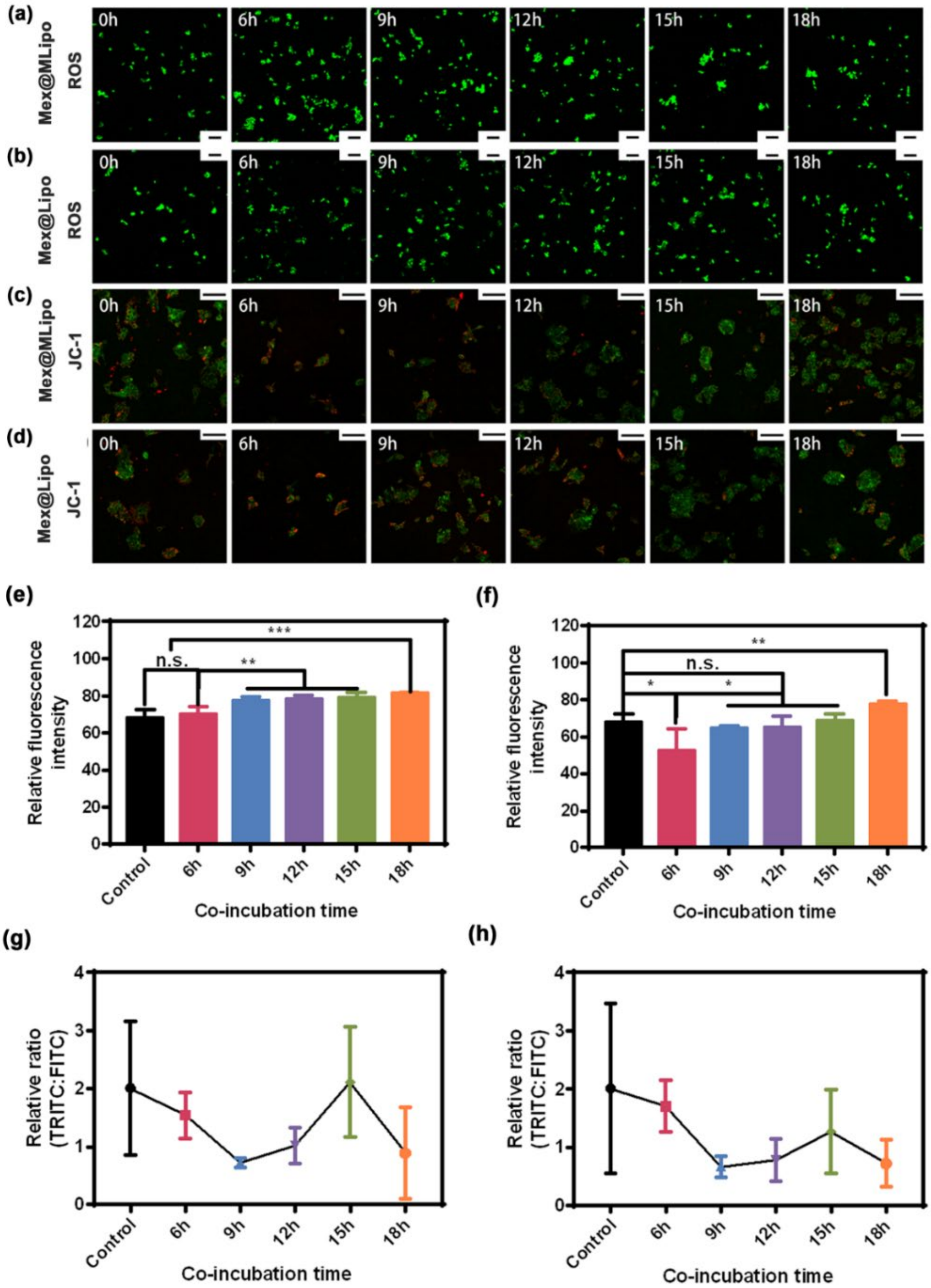


**Figure S6.** Images of intracellular ROS fluorescence of HepG-2 cells after co-incubation with Mex@MLipo (**a**) and Mex@Lipo (**b**) and quantitative analysis of fluorescence intensity respectively (**e**,**f**); Images of JC-1 fluorescence of HepG-2 cells after co-incubation with Mex@MLipo (**c**) and Mex@Lipo (**d**) and quantitative analysis of fluorescence intensity respectively (**g**,**h**). All scale bars: 100 μm. Error bars: mean ± SD (n = 5). The statistical significance is indicated by n.s.: not statistically significant, $0.0001 < {}^{***}p \leq 0.001$, $0.001 < {}^{**}p \leq 0.01$ and $0.01 < {}^{*}p \leq 0.05$, using an unpaired Student's t-test (two-tailed).

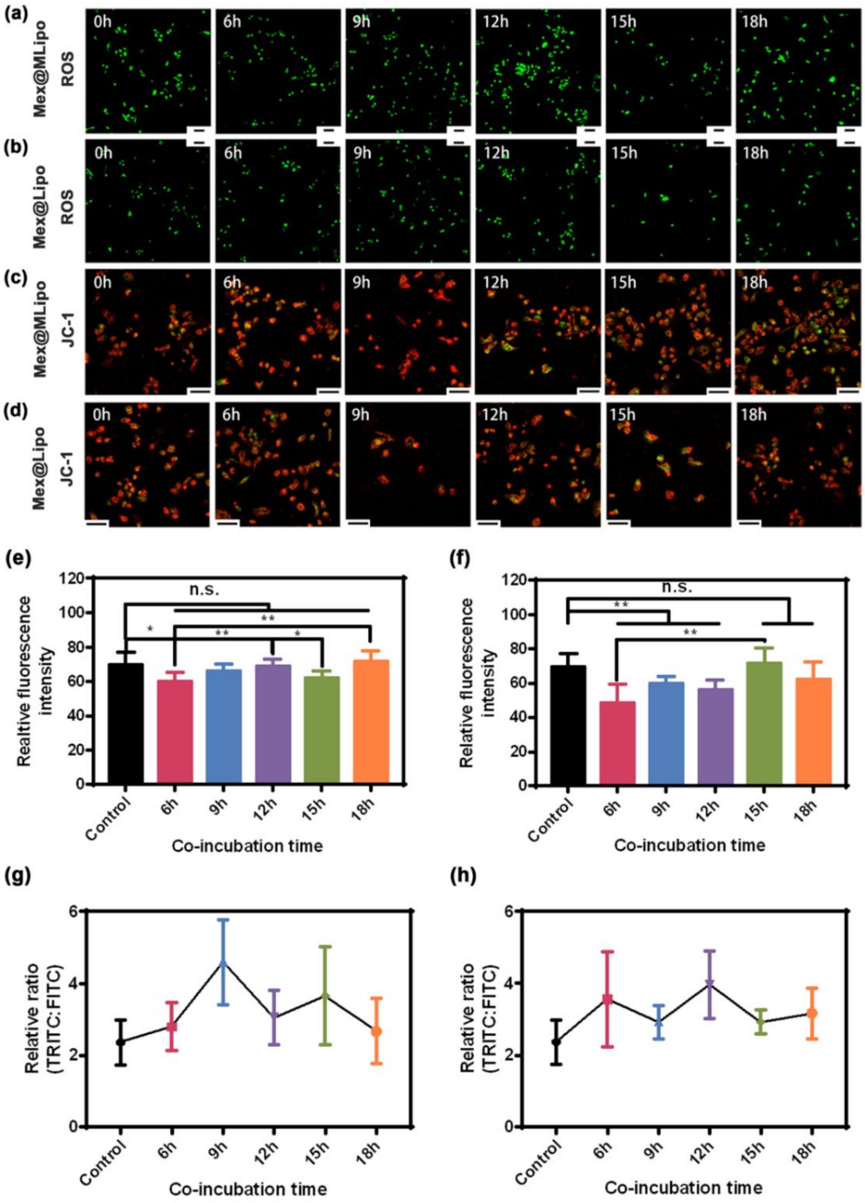


**Figure S7.** Images of intracellular ROS fluorescence of PC-3 cells after co-incubation with Mex@MLipo (**a**) and Mex@Lipo (**b**) and quantitative analysis of fluorescence intensity respectively (**e**,**f**); Images of JC-1 fluorescence of PC-3 cells after co-incubation with Mex@MLipo (**c**) and Mex@Lipo (**d**) and quantitative analysis of fluorescence intensity respectively (**g**,**h**). All scale bars: 100 μm. Error bars: mean ± SD (n = 5). The statistical significance is indicated by n.s.: not statistically significant, $0.001 < {}^{**}p \leq 0.01$ and $0.01 < {}^{*}p \leq 0.05$, using an unpaired Student's t-test (two-tailed).

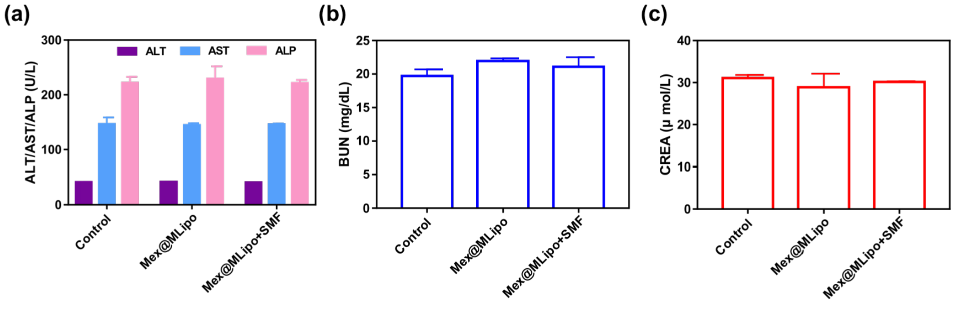


**Figure S8.** (**a**) Serum levels of ALT, AST and ALP in the different treatment groups. (**b**) BUN and (**c**) CREA levels in different treatment groups of mice.
